# Effect of Size and Drying Time on the Rehydration and Sensory Properties of Freeze-Dried Snails (*Achatina achatina*)

**DOI:** 10.1155/2020/5714140

**Published:** 2020-02-11

**Authors:** Matthew A. Achaglinkame, Eric Owusu-Mensah, Abena A. Boakye, Ibok Oduro

**Affiliations:** ^1^Department of Food Science and Technology, Kwame Nkrumah University of Science and Technology (KNUST), Kumasi, Ghana; ^2^Quama Food Pro. Co. Ltd., Kumasi, Ghana

## Abstract

Snails, a delicacy in most tropical communities, are highly perishable and seasonal. Employed preservative methods are highly temperature dependent, adversely affecting their nutritional value and sensory properties. This study was aimed at determining the effect of size and drying time on the rehydration and sensory properties of freeze-dried snails. Snails were sized into three categories with average weights: 7.59 g (quarter-sized), 14.41 g (half-sized), and 30.71 g (whole), and freeze-dried for 15, 20, and 25 h. The moisture content and percent rehydration of the dried samples were determined by standard methods and sensory properties assessed by an in-house panel of 30 using a 5-point hedonic scale. The moisture content of the fresh and freeze-dried samples ranged from 65.80 to 75.20% and 3.25 to 10.24%, respectively. Freeze-dried samples had higher percent rehydration (27 to 102%) than the control; smoked snails (21 to 32%). Size had a significant (*P* < 0.05) effect on the rehydration ability of the samples with the half-sized and freeze-dried for 15 h samples having the highest. The freeze-dried samples generally had higher consumer preference than the control in all attributes assessed. The findings show that freeze-drying snails (approximate weight of 14.4 g) for 15 h could be a consumer-preferred alternative preservative method for extending the shelf life of snails.

## 1. Introduction

Snails are soft-bodied shell-bearing invertebrates which form the largest group of molluscs and the second largest animal group after arthropods [1–3]. They are delicacies in many tropical areas, especially Africa, serving as relatively cheaper alternative protein sources. In terms of world statistics, Morocco, Spain, Indonesia, and China have been reported to be the leading producers of snails while Morocco, Spain, France, and Italy are the leading consumers [1, 4, 5]. In Ghana, the most common species are *Archachatina marginata*, *Archachatina degneri*, *Achatina achatina*, and *Achatina fulica*: *Achatina achatina* is the most preferred [2]. Snail meat is rich in protein (about 80.9–89.92% on dry basis) and low in fat and cholesterol [6, 7] positioning it as a good protein source for health-conscious consumers. The meat of the snail is characteristically tender and chewy, with a unique pleasant floral-like, mushroom-like flavour when boiled [1, 8].

The major limitation to its use however is its high perishability and seasonality [9, 10]. It is reported that the fresh meat of the snail hardly lasts for a day before deteriorating [11]. The land snails are more prone to rapid spoilage as a result of exposure to different kinds of microbes and contaminants as they crawl on the soil [11]. To curb this, they are smoke-dried and strung on sticks for sale [9]. Although the smoked samples have a good market locally, the products do not meet standards for international markets, limiting their exploitation on the export market to supply the needs of locals in the diaspora and to earn foreign exchange for the country.

Several high-temperature drying methods such as oven drying, tray drying, and solar drying have been employed in an attempt to preserve and extend the shelf life of snail meat [6, 7, 9]. These methods, however, do not yield good sensorial and nutritional properties of the snail meat [7, 9] necessitating the need to explore other drying methods which involve low temperatures.

Freeze-drying is a low-temperature drying method that is widely acclaimed for drying heat-labile products such as quality food, pharmaceutical and biomedical products [10]. Freeze-dried products have been reported to have negligible shrinkage, more porous structure, better sensory attribute retention, and improved rehydration ability compared to products dried by other methods [10, 12]. However, there is limited data on the potential use of the technology as an alternative to snail drying and the impact of the technique on the quality parameters of the dried snails. This study therefore investigated the effect of size and drying time on quality (rehydration and sensory) properties of freeze-dried snails to help determine the potential of freeze-drying in the preservation of snail meat.

## 2. Materials and Methods

### 2.1. Sample Collection and Study Location

Sixty medium-sized (about 35 g each) live snails (*Achatina achatina*) and smoked ones (control) were obtained from local markets in Kumasi, Ghana. The instrumental tests and sensory evaluations were carried out at the International Center for Potato Laboratory, Kumasi, Ghana.

### 2.2. Experimental Design

**Figure 1:**
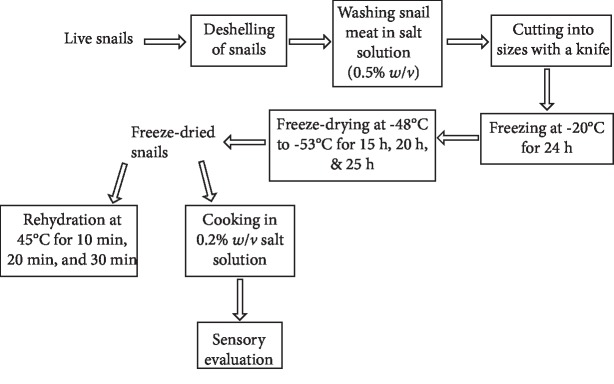
Process flow chart for snail sample preparation, freeze-drying, rehydration, and sensory evaluation.

A 3 × 3 factorial design was employed for this study. Two factors, size of the snail and freeze-drying time, were considered with each having three levels. Size levels included quarter, half, and whole snails while freeze-drying times 15 h, 20 h, and 25 h were employed. The freeze-dried snails were then compared with smoked snails (commercially available dried snails) purchased from the market. Moisture content, rehydration capacity, and sensory properties were the response variables measured. Apart from sensory evaluation, all other analyses were done in triplicate. A diagrammatic flow chart of the study is shown in Figure 1.

### 2.3. Sample Preparation

The snails were deshelled, and the gut content was removed with a stainless steel kitchen knife. The fresh meat was then washed with 0.2% salt solution to remove slime and rewashed with potable water to remove the salt [6]. Three different sizes of the washed meat were used: whole (30.71 ± 1.97 g), half-sized (14.41 ± 0.92 g), and quarter-sized (7.59 ± 0.04 g). The half- and quarter-sized portions were appropriately obtained using a stainless steel knife. Five snails of each size category (whole, half, and quarter) were selected as replicates for further studies.

#### 2.3.1. Freeze-Drying

The snail samples were frozen at -20°C for 24 hr to condition them for the freeze-drying process. The frozen samples of each size were then placed in different plastic bowls of equal size and shape and loaded unto the same shelves in the freeze dryer (YK-118 Vacuum Freeze Dryer). Drying was undertaken at -48 to -53°C for different times: 15, 20, and 25 hours.

### 2.4. Analytical Methods

#### 2.4.1. Moisture Determination

A sample of each of the sizes of the freeze-dried samples was randomly selected and ground. Two grams of each of the powdered samples was oven-dried at 105°C for three hours to determine the residual moisture content (%) of the snail samples freeze-dried for the different times [13] as shown in Equation (1). The moisture content (%) of the fresh snails was also determined by the oven method prior to drying (Equation (1)).

(1) $$\begin{array}{c} \text{Moisture content }\left(\%\right)=\frac{W_{\text{B}}-W_{\text{A}}}{W_{\text{B}}}\times 100, \end{array}$$

where *W*_B_ is the weight (g) of the sample before drying and *W*_A_ is the weight (g) of the sample after drying.

### 2.5. Rehydration Tests

The method as described by Tettey et al. [9] was employed. Triplicates of the freeze-dried snail samples and the control (smoked samples) were rehydrated in 250 ml water at 45°C for thirty minutes at 10 min intervals in a water bath. The rehydration was to help determine the dried snails' ability to imbibe water during cooking. It was also to help screen the products for sensory evaluation. The surface water on the rehydrated sample at each interval upon removal from the water was wiped off with a soft tissue and the rehydrated snail weighed. Percentage rehydration was calculated as described by Tettey et al. [9] with the equation,

(2) $$\begin{array}{c} \text{Rehydration }\left(\%\right)=\frac{W_{\text{r}}-W_{\text{b}}}{W_{\text{b}}}\times 100, \end{array}$$

where *W*_r_ is the weight (g) of the sample after rehydration and *W*_b_ is the weight (g) of the sample before rehydration.

Rehydration curves were plotted from the data, and the freeze-dried samples from each size category that had the highest percent rehydration were selected for the sensory studies.

### 2.6. Sensory Evaluation

An in-house consumer panel of 30 regular snail eaters [14] was used to evaluate the organoleptic properties of the rehydrated snails. Attributes assessed were colour, aroma, taste, chewability, juiciness, and overall acceptability. Commercial smoked snail samples were used as the control. To enhance the perception of sensory attributes of the rehydrated samples, 0.2% salt solution was used for cooking all samples. Cooking was done for approximately 20 min (until meat was tender). The cooked samples were coded and presented in smaller sizes to the panel for evaluation. A five-point hedonic scale (1 = dislike extremely; 5 = like extremely) was used. The assessors were each asked to observe, smell, and chew each coded product and indicate his or her degree of likeness or dislikeness as per the scale provided. Before chewing each product, the assessors rinsed their mouths with water to avoid wrong scaling of the said sensory properties due to carryover effect. The products were then evaluated in separate sensory boots by each assessor to avoid interaction and influence from the other assessors.

### 2.7. Statistical Analysis

SPSS (version 21, 2009) was employed for all analyses. The moisture and rehydration data obtained from the study were analyzed using the general linear model (two-way analysis of variance) while one-way analysis of variance was used for the sensory data at 95% confidence level.

## 3. Results and Discussion

### 3.1. Moisture Determination

The moisture content of the fresh snails ranged from 65.8 to 75.2% with an average of 70.7%. The residual moisture contents of the dried snails are shown in Table 1. It was observed that the moisture contents of the samples decreased with increase in freeze-drying time but increased with increase in sample size. In other words, more moisture was lost in each of the categorized samples as freeze-drying time lengthened which is in agreement with findings by Abasi et al. [15]. Statistically, there was no significant difference between the samples dried for 15 h and those dried for 20 h (*P* > 0.05). However, the 15 h and 20 h freeze-dried samples significantly varied from those freeze-dried for 25 h (*P* < 0.05).

In terms of size, all the samples were statistically similar except for the 15 h sample where the quarter-sized and half-sized samples differed from whole-sized samples. The observed increase in moisture content with increase in sample size indicates that more moisture was lost from the smaller-sized samples as compared to the larger-sized ones. This observation could well be attributed to the surface area of the sample exposed to the drying conditions. That is, the smaller the size of the material, the larger its exposed surface area to the drying conditions and the more moisture is lost from it and vice versa [16]. The moisture of the smoked samples was 8.91% which was relatively higher than that recorded from the freeze-dried samples with the exception of whole-sized samples dried for 15 h.

The low moisture contents of the samples largely translate to low water activity [17]. These lower moisture values suggest more stability and longer storability as little or no free moisture might be available for microbial activity [18].

### 3.2. Rehydration

**Figure 2:**
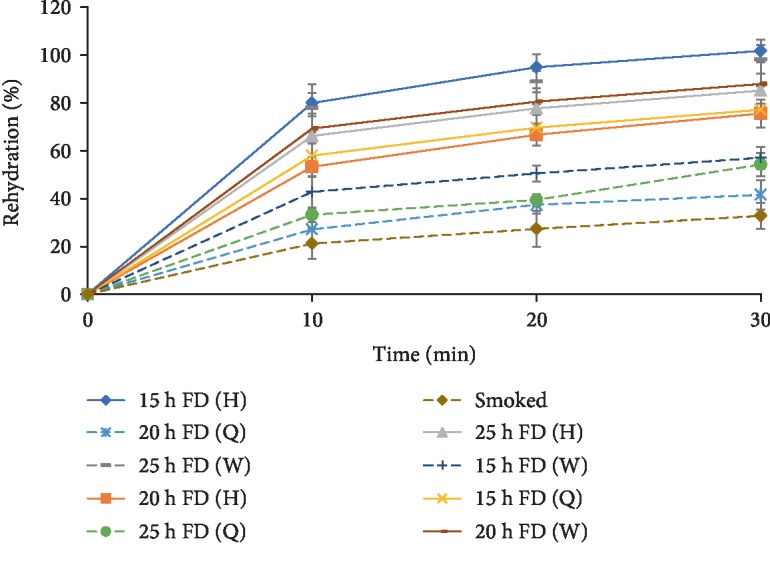
Percentage rehydration of dried snails against time (min). The error bars represent standard deviation. FD: freeze-dried snails; H: half-sized snails; Q: quarter-sized snails; W: whole snails.

It was observed that percentage rehydration increased with rehydration time for all the samples (Figure 2). The percentage rehydration increased sharply during the first ten minutes after which it increased gradually in the remaining period of rehydration. The freeze-dried snail samples generally had higher percentage rehydration (27 to 102%) than the smoked samples (21 to 32%), a good indication of the product's ability to take up water during reconstitution, compared to the smoked ones. Aksoy et al. [19] also obtained similar findings in minced meat where the freeze-dried samples outperformed the ultrasound-assisted vacuum-dried and vacuum-dried samples. The relatively higher rehydration capacity of freeze-dried samples as compared to samples dried by other methods has been linked with higher porosity in freeze-dried samples than the others [10, 12]. Freeze-drying has also been reported to enhance connectivity of the pore structure of the sample to enable faster rehydration [20]. These characteristics associated with freeze-drying may best explain findings in this study.

It was also observed that among the freeze-dried samples, the 15 h dried snails had the highest percentage rehydration in both the quarter- and half-sized categories while the 25 h samples had the highest percentage rehydration for the whole-dried samples. The shorter drying time but high percentage rehydration for quarter- and half-sized samples indicates faster reconstitution and thus potentially shorter cooking time and less energy consumption. It was however observed that smaller-sized samples dried for 20 and 25 h had relatively lower percentage rehydration compared to their corresponding whole samples. Overdrying is said to cause rupture or collapse of cells [16], and this could best explain the low percentage rehydration in the smaller-sized samples dried for 20 h and 25 h. Findings from this study also showed that the 15 h freeze-dried whole samples had a relatively lower percentage rehydration than those dried for 20 and 25 h, which could be attributed to the high moisture content (10.24%) of the 15 h freeze-dried whole samples (Table 1).

### 3.3. Sensory Evaluation

**Figure 3:**
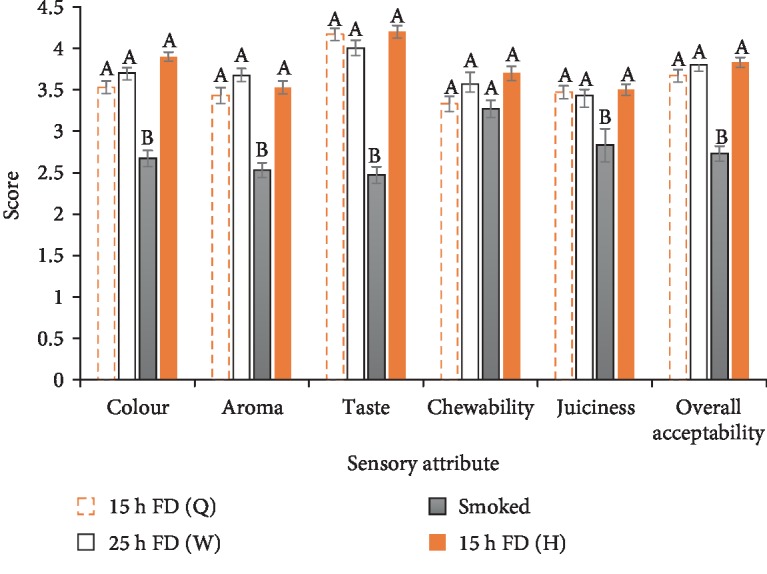
Sensory properties of cooked freeze-dried and smoked snails. Bars with the same superscript under the same attribute are not significantly different from each other. The error bars represent standard deviation. FD: freeze-dried snails; H: half-sized snails; Q: quarter-sized snails; W: whole snails.

Generally, the freeze-dried samples were more preferred to the smoked samples in all the attributes assessed (Figure 3). This affirms earlier reports that freeze-drying preserves sensory properties of products because of the low temperatures involved [8]. It was also observed that the 15 h freeze-dried half-sized samples were the most liked in all the sensory attributes assessed, except for aroma where the 25 h freeze-dried whole samples were the most liked. This is an indication that freeze-drying half-sized snails for 15 h may not only give good rehydration properties but also maintain their organoleptic properties. However, there were no statistical differences (*P* > 0.05) between the freeze-dried samples for all assessed attributes although each was significantly different (*P* < 0.05) from those of the smoked snails except for chewability.

It has been reported that deposits of smoke particles on samples adversely affect sensory properties of the samples [5, 6]. This may have been a possible cause for the low scores (inferred from the low panel preference) in sensory quality of the smoked samples, further positioning freeze-drying as a more suitable alternative for snail drying. The relatively high consumer preference for the freeze-dried snails also infers better market potential than smoked snails.

## 4. Conclusions

The study revealed that size has a significant effect on the rehydration ability of freeze-dried snails but not on their sensory properties. Findings of this study showed that freeze-dried snails have better rehydration and sensory properties than smoked snails. It therefore suggests that freeze-dried snails may have better market potential than smoked snails. Moreover, freeze-drying enhanced the rehydration rate of the dried snails. Smaller-sized samples (7 to 15 g) should be used and freeze-drying done for 15 h for better rehydration.

## Figures and Tables

**Table 1:** Moisture content (%) of freeze-dried and smoked snails.

Snail Size (fresh weight/g)	% moisture (freeze-dried snails)			% moisture of commercial smoked snail (control)
	15 h	20 h	25 h	
Quarter (7.59 ± 0.04)	6.47 ± 0.05^aA^	5.47 ± 0.51^aA^	3.25 ± 0.03^bA^	
Half (14.41 ± 0.92)	7.00 ± 0.05^aA^	5.97 ± 0.04^aA^	3.48 ± 0.02^bA^	
Whole (30.71 ± 1.97)	10.24 ± 1.34^aB^	7.43 ± 0.24^aA^	4.48 ± 0.63^bA^	8.91 ± 0.94

Values (mean ± standard deviation) with the same superscript in the same row or column are not significantly different from each other. The lowercase letters compared values in the same row in terms of the same size across drying time while the uppercase letters compared values in the same column in relation to the different sizes under the same drying time. 15 h, 20 h, and 25 h were the drying times used.

## Data Availability

The data used to support the findings of this study are available from the corresponding author upon request.
